# Quality of Life in Patients with Endodontically Treated Teeth: A Retrospective Study at an Educational Hospital

**DOI:** 10.3390/healthcare12222248

**Published:** 2024-11-11

**Authors:** Rahaf A. Almohareb, Reem M. Barakat, Hadeel M. Basuhail, Shahad A. Alshihri, Nada Y. Alturki, Rafa Alsultan, Ghadah T. Alrashid, Ghadeer Y. Alotaibi, Mamata Hebbal

**Affiliations:** 1 Department of Clinical Dental Sciences, College of Dentistry, Princess Nourah Bint Abdulrahman University, P.O. Box 84428, Riyadh 11671, Saudi Arabia; raalmohareb@pnu.edu.sa (R.A.A.); hbasuhail99@gmail.com (H.M.B.); shahadahmad847@gmail.com (S.A.A.); 439002444@pnu.edu.sa (N.Y.A.); 439001223@pnu.edu.sa (R.A.); alghada456@gmail.com (G.T.A.); ghadeer.alotaibii4@gmail.com (G.Y.A.); 2 Dental Clinics Department, King Abdullah bin Abdulaziz University Hospital, Princess Nourah Bint Abdulrahman University, P.O. Box 84428, Riyadh 11671, Saudi Arabia; 3 Department of Preventive Dental Sciences, College of Dentistry, Princess Nourah Bint Abdulrahman University, P.O. Box 84428, Riyadh 11671, Saudi Arabia; mihebbal@pnu.edu.sa

**Keywords:** endodontics, oral-health-related quality of life, patient satisfaction, periapical index, root canal treatment

## Abstract

Background/Objectives: Root canal treatment (RCT) is a crucial procedure for preserving dental health. While its clinical success rates are well documented, patient-reported outcomes regarding quality of life remain less explored. This study aimed to assess the long-term impact of RCT performed in an educational hospital on patients’ oral-health-related quality of life (OHRQoL) using the Oral Health Impact Profile-14 (OHIP-14). Methods: A total of 1500 patients who underwent non-surgical RCT between April 2018 and February 2023 were called and invited for a follow-up visit. During the visit, all teeth that had undergone RCT were assessed clinically and radiographically by two calibrated examiners to evaluate RCT quality and pre- and post-treatment periapical index (PAI) scores. Demographic information and OHIP-14 responses were collected, and the data were recorded. Results: Patients reported high levels of satisfaction (95%) and no negative impact on their OHRQoL. Statistical analysis revealed that post-operative periapical index (PAI) scores (*p* < 0.001), patient gender (*p* = 0.003) and nationality (*p* = 0.029) significantly influenced OHRQoL perceptions; Conclusion: These findings emphasize the effectiveness of RCT in enhancing patients’ quality of life and highlight the impact of demographic factors—such as gender and whether the patient is a resident or a national of the country, along with post-treatment severity of the disease—on patient-reported outcomes.

## 1. Introduction

Root canal treatment (RCT) stands as a cornerstone of modern dentistry, dedicated to preserving natural dentition and restoring oral health. It encompasses therapeutic interventions aimed at addressing diseases and infections affecting the dental pulp and periapical tissues [1]. The primary objective of RCT, also known as endodontic treatment, is to prevent or treat periapical infection (apical periodontitis), thereby alleviating pain and maintaining the functionality of the affected tooth [2]. RCT targets infections whose main pathogen is a complex oral bacterial biofilm. It involves cleaning and shaping the complex root canal system of a tooth using chemical and mechanical techniques that can neutralize this biofilm and prepare the canal to receive a filling material that seals it from future bacterial invasion [3,4,5].

Direct assessment of the root canal and periapical tissues to determine whether the infection has been successfully eliminated is challenging due to their inaccessibility. Consequently, multiple concepts have been proposed to define RCT outcomes. For one, “success” is evaluated clinically and radiographically by absence of any radiographic sign of apical periodontitis with a concomitant lack of any clinical signs and symptoms. RCT boasts high success rates ranging between 82% and 92.6% [6,7]. However, these outcomes provide little insight into patients’ perceptions and experiences following treatment. They also fail to assess the broader impact such procedures have on a patient’s well-being, such as social confidence, psychological well-being related to fear and anxiety of dental procedures and its effect on daily activities like eating, speaking, and smiling.

Oral-health-related quality of life (OHRQoL) is a concept that measures how oral health influences overall well-being, encompassing functional limitations, pain, psychological discomfort, and social and emotional impacts [8,9]. Studies underscore the significant link between good OHRQoL and overall quality of life [10,11,12,13].

Healthcare providers have several tools for assessing OHRQoL, one of which is the Oral Health Impact Profile (OHIP) questionnaire. It consists of 49 items, which were later simplified into a shorter version comprising 14 items, known as the Oral Health Impact Profile-14 (OHIP-14) [14]. The OHIP-14 has proven to be sensitive to measuring response to endodontic treatment, making it a valuable tool for understanding patients’ perspectives on the outcomes of their endodontic care [15].

Despite existing research using the OHIP-14 to evaluate short-term impacts of RCT [1], there remains a gap in understanding long-term effects and specific factors influencing OHRQoL, particularly in Saudi Arabia. A patient’s OHRQoL reflects their goals, expectations, standards, and concerns within the context of their cultural conditions and value system [16]. Existing literature from the region primarily focuses on clinical outcomes rather than patient-centered assessments [17]. Therefore, this study aims to assess the OHRQoL of patients who have undergone RCT at an educational hospital in Riyadh, Saudi Arabia, using the OHIP-14 tool, and to identify the influencing factors.

## 2. Materials and Methods

Ethical review and approval were waived for this cross-sectional study by the Institutional Review Board at Princess Nourah Abdul Rahman University (PNU IRB # 23-0563) on 6 August 2023.

### 2.1. Study Setting and Eligibility Criteria

The study was conducted at the PNU dental clinics among patients with records of non-surgical RCT from 2018 to February 2023, as documented in the AxiUm electronic health records system (Exan, Henry Schein, NV, USA). Inclusion criteria required patients to be aged 18 years or older; have complete data in AxiUm, including clear pre- and post-operative periapical radiographs; and have attended a recall visit at least six months post-RCT (Table 1).

As per the clinical guidelines of PNU Dental Clinics, rubber dam isolation is mandatory for all non-surgical root canal treatments. Canal preparation involves instrumentation using a combination of ‘manual and rotary instruments. Irrigation is carried out with 5.25% sodium hypochlorite, followed by saline as the final rinse. Cases are distributed according to the American Association of Endodontics Case Difficulty Assessment among undergraduate dental students, interns, endodontic board residents, and endodontists. All the students, interns, and board residents work under the close supervision of an endodontist following the standard protocol.

A total of 1500 patients meeting the eligibility criteria were contacted and offered recall appointments. During the appointment, the patients’ consent to participate in the study was obtained after the study’s purpose was explained. Patients were informed that participation was voluntary, and that refusal would not affect their treatment at PNU dental clinics. Each patient underwent a thorough clinical and radiographic examination and was then asked to complete the OHIP-14 questionnaire via Google Forms [18].

During recall appointments, patients underwent comprehensive clinical and radiographic examinations conducted by two calibrated examiners. The periapical index (PAI), a validated five-point scale, was used to assess periapical healing. Two calibrated examiners conducted both clinical and radiographic assessments to evaluate the quality of RCT and the pre- and post-treatment PAI scores. Data were recorded and coded in an Excel sheet, with each tooth evaluated as a single unit. Tooth position in the arch and the operator performing treatment were also recorded. Operators were divided into five categories: undergraduate student, intern, general practitioner, endodontic board resident, and endodontist.

### 2.2. Questionnaire Structure

The questionnaire consisted of 3 parts totaling 31 closed-ended questions. Part 1 included demographic data (age, sex, nationality, level of education, income, occupation, and health condition) and questions related to oral health status. Part 2 adapted the Oral Health Impact Profile (OHIP) into the OHIP-14 Arabic version, which consisted of 14 questions covering seven dimensions: functional limitation, physical pain, psychological discomfort, physical disability, psychological disability, social disability, and handicap [1]. The translation process involved initial translation from English to Arabic by a bilingual expert and subsequent back-translation to ensure content validity. A review committee approved the final Arabic version after testing for comprehension among six volunteers not involved in the study. Part 3 assessed participants’ general perception regarding RCT with seven additional questions (Table 2). To assess reliability, the questionnaire was administered to 12 separate volunteers initially and again after an interval of eight days. The consistency of responses over this period was analyzed to establish internal reliability.

### 2.3. Reliability and Calibration

Inter-rater and intra-rater reliability for PAI scoring and RCT quality evaluation were assessed using Cohen’s kappa statistics. Calibration for PAI scoring achieved a Cohen’s kappa of 0.69, indicating acceptable agreement, while intra-observer agreement reached a Cohen’s kappa of 0.85. Inter- and intra-operator agreement for RCT quality evaluation ranged between 0.836 and 1, indicating excellent agreement.

### 2.4. Sample Size

A minimum sample size was calculated based on a 90% expected frequency of satisfaction among patients attending PNU Dental Clinics and an allowable error of 4%. The minimum sample size required was found to be 216. This was also based on previous studies [21]. The sample size in this study exceeds this minimum required sample size, which significantly enhances the study’s statistical power.

### 2.5. Statistical Analysis

Since the two extreme values in the 5-point Likert scale used in the OHIP-14 part of the questionnaire were minimal, they were combined into a 3-point scale. Statistical analyses were performed using SPSS software (version 28, SPSS Inc., Chicago, IL, USA). Associations between demographic variables, endodontic tooth status, and OHRQoL were analyzed using the chi-squared test, with significance set at *p* < 0.05.

## 3. Results

A total of 245 patients participated in the study, comprising predominantly females (67%) and Saudi nationals (80.7%). The majority of participants reported a low income (73.8%), were in good health (73%), and were non-smokers (81%) (Table 3).

**Figure 1:**
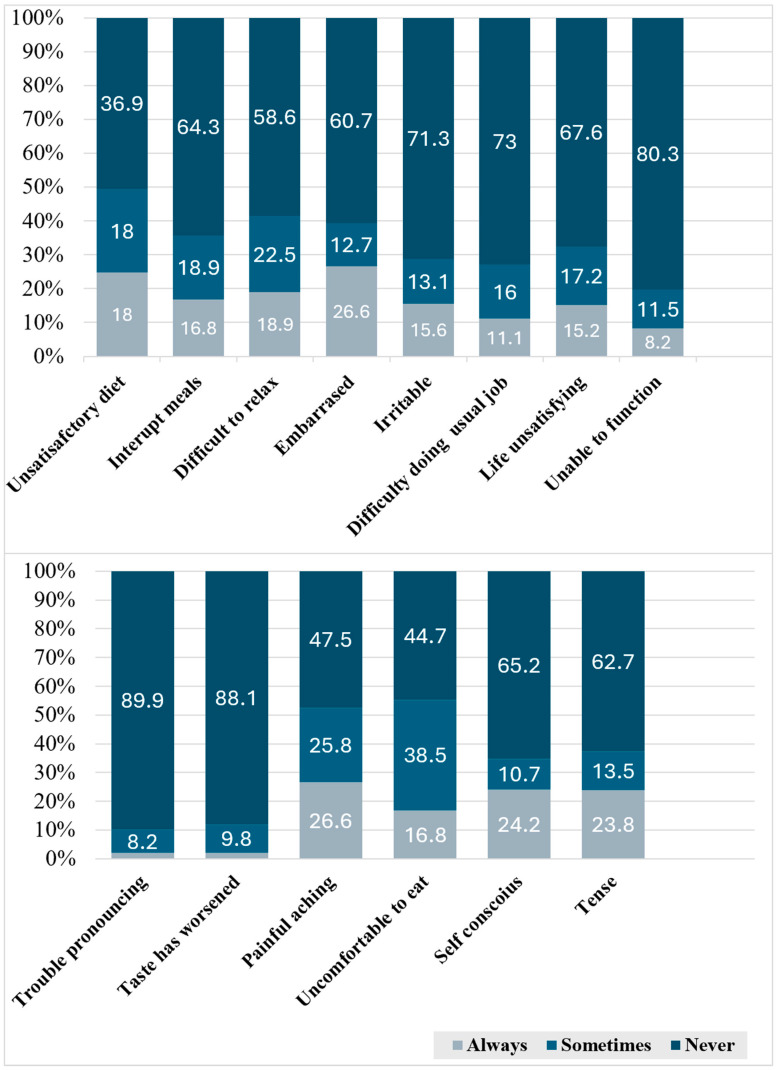
Frequencies of patient responses to the Modified OHIP-14 Questionnaire, detailing treatment impact across various domains of oral-health-related quality of life (OHRQoL).

Post-endodontic treatment satisfaction was high, with 95.1% of patients expressing satisfaction and 95.9% recommending the procedure to others. The majority of patients (60.7%) reported that their experience of root canal treatment was better than expected. A significant portion of patients (60.7%) reported that their experience with root canal treatment exceeded their expectations. While 45.9% experienced pain during treatment, only 12.3% cited it as a source of dissatisfaction afterward. The mean OHRQoL score was 35.12 ± 5.57 (Figure 1). There was no significant correlation between OHRQoL scores and tooth position (*p* = 0.659) nor the operator performing the treatment (*p* = 0.203).

Significant correlations were observed between OHRQoL scores and demographic factors. Specifically, OHRQoL scores were significantly associated with patient gender (*p* = 0.003), with males reporting lower scores. Saudi nationality (*p* = 0.029) and lower educational levels (*p* = 0.014) were also associated with lower OHRQoL scores (Table 4).

## 4. Discussion

Although pulpal pathology is a common cause of dental pain, research on patients’ long-term views of root canal treatment (RCT) remains limited. This cross-sectional study aimed to explore patient experiences and perceptions of RCT, focusing on individuals who underwent the procedure between 6 months and 6 years ago.

The results demonstrated that root canal therapy significantly enhanced patients’ quality of life, with 95% expressing satisfaction and nearly 96% recommending the procedure to others. These findings align with previous cross-sectional and longitudinal studies that reported improved oral-health-related quality of life (OHRQoL) scores following root canal treatment [15,22,23]. A recent systematic review evaluating the impact of endodontic treatment on quality of life found that both non-surgical primary RCT and retreatment generally improved patients’ quality of life [24]. However, these results are limited to patients seeking endodontic treatment and may not be generalizable to all populations [1].

The present study’s findings are consistent with a recent four-year follow-up study that observed an improvement in oral-health-related quality of life (OHRQoL) post-treatment [25]. Similarly, Sanz et al. (2022) reported high long-term patient satisfaction two years after root canal treatment performed by experienced practitioners [26].

Gender was found to influence patients’ perceptions, with females reporting significantly poorer OHRQoL compared to males. While some studies have found no significant influence of gender on OHRQoL [27,28,29,30], previous Scandinavian epidemiological studies have also noted poorer OHIP-14 scores in females compared to males [31].

OHRQoL measures are inherently subjective and reflect the expectations of individuals who have adapted to specific life situations or local environments [32]. Although research has shown that OHRQoL is associated with patients’ socioeconomic status [33,34], the present study found that patients’ perceptions of the effect of RCT on their OHRQoL were not related to their income or educational level. In contrast, patient nationality did impact OHRQoL scores after RCT, with citizens reporting a higher level of satisfaction compared to non-citizens. Nationality and cultural background play a complex role in shaping healthcare experiences. Research indicates that when patients and healthcare providers share similar backgrounds, it often leads to more positive patient experiences and higher satisfaction with the healthcare provided [35].

Other studies have identified older age and smoking status as factors associated with poorer OHRQoL [28]. It is important to note that these studies were conducted over shorter durations following endodontic treatment. A longitudinal study evaluating the OHRQoL of 250 patients at various time intervals concluded that there is a strong relationship between disease state and OHRQoL [36]. This finding is echoed in the present study, where post-operative PAI scores were significantly correlated with OHRQoL scores. However, the presence of clinical signs and symptoms did not affect patients’ OHRQoL, which contradicts previous findings [25]. Additionally, the duration since root canal treatment was found to influence OHRQoL differently compared to other procedures [37]. While the present study focused on exploring OHRQoL after an extended period post-treatment, many studies measure OHRQoL only shortly after treatment (ranging from a week to several months).

Consistent with previous studies [23,30], the two main OHRQoL domains negatively impacted were psychological discomfort (embarrassment) and physical pain, affecting 26% of patients. Additionally, nearly 17% reported discomfort while eating (Figure 1). These levels were significantly lower than those reported in earlier studies and did not affect overall patient satisfaction with RCT. It is important to note, however, that the sample in this study comprised only patients who attended a follow-up visit. The presence of physical pain may have motivated these patients to return for follow-up, unlike those who did not attend.

Previous studies have explored the impact of dental treatment in general and RCT in particular on OHRQoL in Saudi Arabia [38]. These studies typically followed patients for a short term post-RCT (ranging from one week to six months) [28,39]. Consistent with the findings of this study, satisfaction with RCT was high, highlighting physical pain as the most affected dimension. However, different factors, such as income level and age, were associated with this satisfaction. A controlled clinical trial also found that both single- and multiple-visit RCT improved OHRQoL [17].

According to a previous systematic review, the impact of different operator types (endodontist, postgraduate student, general practitioner, and dental student) on quality of life following endodontic treatment was inconclusive [24]. Conversely, meta-regression analyses from a recent systematic review indicated that operators’ qualifications did not significantly impact the success rates of RCT [6]. In the current study, OHRQoL scores were not influenced by the type of operator performing the treatment. This may be due to less experienced operators, such as students, interns, and residents, performing RCTs under the close supervision of an experienced endodontist. Additionally, there may be potential bias, as endodontists tend to handle more complex cases compared to those treated by general dentists and undergraduate dental students.

The position of the tooth in the arch did not significantly influence OHRQoL scores, which is consistent with findings from a previous study [40]. While another study identified tooth type as a predictor of certain discomforts and limitations [41], patients’ perceptions of their overall well-being and satisfaction after treatment may be more strongly influenced by the psychological relief associated with successful treatment, which can outweigh any functional differences related to the tooth’s position.

A key limitation of this study is the low participation rate (16.6%), which may indicate potential response bias, as patients with positive experiences might be more motivated to attend follow-up visits and participate. Additionally, the retrospective design meant that OHRQoL was only measured post-RCT, preventing comparisons with pre-treatment levels and limiting the ability to establish cause-and-effect relationships. While the study provides insights into OHRQoL after RCT, it does not directly link improvements to the treatment itself. Furthermore, focusing on a single educational hospital may restrict the generalizability of the findings to other healthcare settings. Future well-designed longitudinal studies with larger sample sizes are needed to assess the applicability of these results across diverse populations. Further research comparing long-term patient-centered outcomes between various endodontic treatment options—both surgical and non-surgical—and more conservative approaches are also recommended.

## 5. Conclusions

While root canal treatment often induces anxiety, the majority of patients reported high satisfaction levels and no negative impact on their oral-health-related quality of life (OHRQoL) post-treatment. Factors such as patient gender, nationality, and post-operative PAI scores significantly influenced perceptions of RCT and patient-reported outcomes.

## Figures and Tables

**Table 1:** Study inclusion and exclusion criteria.

Domain	Inclusion	Exclusion
**Patient Age**	18 years old and above.	<18 years old. Individuals with psychological conditions or disabilities that hinder their ability to comprehend the purpose of the study.
**Electronic Health Records**	Complete pre- and post-root-canal treatment (RCT) data. Clear pre- and post-RCT periapical radiographs.	One or more missing data.
**Root Canal Treatment (RCT)**	Non-surgical RCT completed between April 2018 and February 2023. RCT on posterior and anterior teeth.	RCT less than six months ago. Surgical root canal treatment.
**Recall visit**	Attended the recall visit post-RCT.	Did not attend the recall visit.

**Table 2:** Questionnaire administered to patients during recall visit.

**Age**	**Income**
18–29	Less than 10,000
30–49	10,000–50,000
50–64	50,000–100,000
65 and older	100,000–150,000
**Gender**	More than 150,000
Male	**Occupation**
Female	Health practitioner
**Nationality**	Engineer
Saudi	Business
Non-Saudi	IT and communication
**Level of education**	Financing
Elementary school	Lawyer
Middle school	Education
High school	Unemployed (Housewife, retired, student, etc.)
Bachelor	Others
Masters	
PHD	
**Health**	
**General health**	
Healthy	
Not healthy	
**If you answered with “Not healthy” please mention the disease/s you have**	
**Smoking status**	
Smoker	
Previous smoker	
Not a smoker	
**Oral hygiene** (Multiple choices)	
I don’t brush my teeth	
I brush once daily	
I brush twice or more daily	
I use dental floss	
I use mouth wash	
**Oral Health Impact Profile**	
Have you had trouble pronouncing any words because of problems with your teeth or mouth?	
Have you felt that your sense of taste has worsened because of problems with your teeth or mouth?	
Have you had any painful aching in your mouth?	
Have you found it uncomfortable to eat any food because of problems with your teeth or mouth?	
Have you been self-conscious because of your teeth or mouth?	
Have you felt tense because of problems with your teeth or mouth?	
Has your diet been unsatisfactory because of problems with your teeth or mouth?	
Have you had to interrupt meals because of problems with your teeth or mouth?	
Have you found it difficult to relax because of problems with your teeth or mouth?	
Have you been a bit embarrassed because of problems with your teeth or mouth?	
Have you been a bit irritable with other people because of problems with your teeth or mouth?	
Have you had difficulty doing your usual job because of problems with your teeth or mouth?	
Have you felt that life in general was less satisfying because of problems with your teeth or mouth?	
Have you been totally unable to function because of problems with your teeth or mouth?	
**Patient perception**	
**Having had root canal treatment, was the experience better or worse than you expected?**	
Worse than expected	
As expected	
Better than expected	
**What dissatisfaction, if any, did you have following root canal treatment? (Multiple choices)**	
No concerns	
Pain associated with the treatment	
Time-consuming	
Needing future treatment or maintenance	
Treatment failure	
Others: ________________________	
**Did you experience any pain during your root canal treatment?**	
Yes	
No	
**If you answered “Yes”, please rate your pain:**	
Not painful	
Slightly painful	
Painful	
Extremely painful	
**After having root canal treatment, how happy are you to have kept your tooth?**	
Very happy	
Indifferent	
I’d rather have my tooth removed	
**After having root canal treatment, would you advise your loved ones to do it?**	
Yes	
No	
**If you had to have root canal treatment again, how would you feel about it?**	
1 (Not nervous at all)	
2	
3	
4	
5 (Very nervous)	
**Overall, are you satisfied with the outcome of your root canal treatment?**	
Yes	
No	

Reproduced with permission from [19,20].

**Table 3:** Descriptive statistics for sample demographics.

Demographic Variables		Frequency	Percentage (%)
**Gender**	Male	80	32.8
	Female	165	67.2
**Income**	Less than 10,000	181	73.8
	10,000–50,000	59	24.2
	50,000–100,000	3	1.2
	More than 100,000	2	0.8
**Education**	High School or Below	84	34.4
	Bachelor	139	56.6
	Master’s	11	4.5
	PhD	11	4.5
**Age**	65 or Older	7	2.9
	50–64	55	22.5
	30–49	101	41.0
	18–29	82	33.6
**Nationality**	Saudi	198	80.7
	Non-Saudi	47	19.3
**Smoking**	Smoker	34	14.0
	Previous Smoker	13	5.0
	Non-Smoker	198	81.0
**Occupation**	Healthcare Practitioner	22	9.0
	Construction (Engineer)	5	2.0
	IT and Communication	12	5.0
	Education	49	20.0
	Financing	20	8.0
	Business	30	12.0
	Lawyer	2	1.0
	Unemployed	105	43.0
**Medical Condition**	Healthy	179	73.0
	Asthma	17	7.0
	Diabetes	30	12.0
	Hypercholesterolemia	2	1.0
	Hypertension	7	3.0
	Hypothyroidism	10	4.0

**Table 4:** Chi-square test results for factors significantly associated with improved oral-health-related quality of life (OHRQoL) Scores.

Variable		*p*-Value
**Demographic**	Age	0.828
	Gender	0.003 *
	Nationality	0.029 *
	Education	0.140
	Income	0.66
	Occupation	0.137
	Smoking	0.589
	Medical condition	0.619
**Tooth related**	Pre-operative PAI Score	0.272
	Tooth	0.937
	Post-operative PAI Score	<0.001 *
	Clinical symptoms	0.517
	Restoration type	0.830
	Restoration material	0.568
	Tooth position in the arch	0.659
**Operator related**	Operator performing treatment	0.204

* Presence of significant association set at *p* < 0.05.

## Data Availability

The data that support the findings of this study are available from the corresponding author upon reasonable request.
